# Prognostic accuracy of the serum lactate level, the SOFA score and the qSOFA score for mortality among adults with Sepsis

**DOI:** 10.1186/s13049-019-0609-3

**Published:** 2019-04-30

**Authors:** Zhiqiang Liu, Zibo Meng, Yongfeng Li, Jingyuan Zhao, Shihong Wu, Shanmiao Gou, Heshui Wu

**Affiliations:** 1 Department of Pancreatic Surgery, Union Hospital, Tongji Medical College, Huazhong University of Science and Technology, Wuhan, 430022 China; 2 Department of Gastrointestinal Surgery, Union Hospital, Tongji Medical College, Huazhong University of Science and Technology, Wuhan, 430022 China

**Keywords:** Sepsis, Lactate, qSOFA, SOFA, Prognosis, MIMIC III

## Abstract

**Background:**

Sepsis is a common critical condition caused by the body’s overwhelming response to certain infective agents. Many biomarkers, including the serum lactate level, have been used for sepsis diagnosis and guiding treatment. Recently, the Third International Consensus Definitions for Sepsis and Septic Shock (Sepsis-3) recommended the Sequential Organ Failure Assessment (SOFA) and the quick SOFA (qSOFA) rather than lactate for screening sepsis and assess prognosis. Here, we aim to explore and compare the prognostic accuracy of the lactate level, the SOFA score and the qSOFA score for mortality in septic patients using the public Medical Information Mart for Intensive Care III database (MIMIC III).

**Methods:**

The baseline characteristics, laboratory test results and outcomes for sepsis patients were retrieved from MIMIC III. Survival was analysed by the Kaplan-Meier method. Univariate and multivariate analysis was performed to identify predictors of prognosis. Receiver operating characteristic curve (ROC) analysis was conducted to compare lactate with SOFA and qSOFA scores.

**Results:**

A total of 3713 cases were initially identified. The analysis cohort included 1865 patients. The 24-h average lactate levels and the worst scores during the first 24 h of ICU admission were collected. Patients in the higher lactate group had higher mortality than those in the lower lactate group. Lactate was an independent predictor of sepsis prognosis. The AUROC of lactate (AUROC, 0.664 [95% CI, 0.639–0.689]) was significantly higher than that of qSOFA (AUROC, 0.547 [95% CI, 0.521–0.574]), and it was similar to the AUROC of SOFA (AUROC, 0.686 [95% CI, 0.661–0.710]). But the timing of lactate relative to SOFA and qSOFA scores was inconsistent.

**Conclusion:**

Lactate is an independent prognostic predictor of mortality for patients with sepsis. It has superior discriminative power to qSOFA, and shows discriminative ability similar to that of SOFA.

**Electronic supplementary material:**

The online version of this article (10.1186/s13049-019-0609-3) contains supplementary material, which is available to authorized users.

## Background

Sepsis is a life-threatening complication of infection and characterized by physiologic, pathologic, and biochemical abnormalities [1, 2]. It is the tenth-most-common cause of death globally [3] and the most common cause of death in patients with infections, especially when sepsis is not identified and treated promptly. Early treatment of sepsis improves patients’ mortality and outcome [1].

Serum lactate level is a sensitive but nonspecific indicator of metabolic stress [4]. As a product of anaerobic glycolysis, lactate is increased during hypoxia, stress and many critical illnesses [5]. Recent research presents a positive association between higher levels of lactate and increased mortality [6]; the higher the lactate level, the worse the outcome [7]. Different lactate thresholds have been recommended in some studies as an early aggressive resuscitation predictor [7–12]. Based on this, early identification of elevated serum lactate levels can potentially lead to early identification of patients who are in danger of poor outcomes. Sequential Organ Failure Assessment (SOFA) [13] and quick SOFA (qSOFA) [1, 14] were designed to measure organ dysfunction in patients admitted to intensive care units (ICU). We further explored the prognostic accuracy of the serum lactate level, the SOFA and the qSOFA in predicting mortality in patients with sepsis.

## Materials and methods

### Data source

The data used in this study were collected from Medical Information Mart for Intensive Care III database version 1.4 (MIMIC III v1.4), a publicly available single-center critical care database. It includes information on 46,520 patients who were admitted to the ICU of Beth Israel Deaconess Medical Center in Boston, Massachusetts from 2001 to 2012 [15]. The database documents included charted events such as demographics, vital signs, laboratory tests, fluid balance and vital status. International Classification of Diseases, Ninth Revision (ICD-9) codes were also documented by hospital staff on patient discharge. Hourly physiologic data from bedside monitors validated by ICU nurses were recorded. Written evaluations of radiologic films by specialists covering in the corresponding time period were stored in the database. The documentation in the database was provided by clinicians, data scientists, information technology personnel and users [15, 16]. The project was approved by the institutional review boards of the Massachusetts Institute of Technology (MIT) and Beth Israel Deaconess Medical Center (BIDMC); there was no requirement for individual patient consent because unidentified health information of patients was used. The raw data were extracted using structure query language (SQL) with Navicat Premium version 12.0.28 and further processed with R software (version 3.4.3, CRAN). The code that supports the MIMIC-III documentation and website is publicly available, and contributions from the community of users are encouraged (https://github.com/MIT-LCP/mimic-website). The codes used to generate the descriptive statistics are included in a notebook that is available at: https://github.com/MIT-LCP/mimic-iii-paper/ [15].

### Data extraction and management

**Fig. 1:**
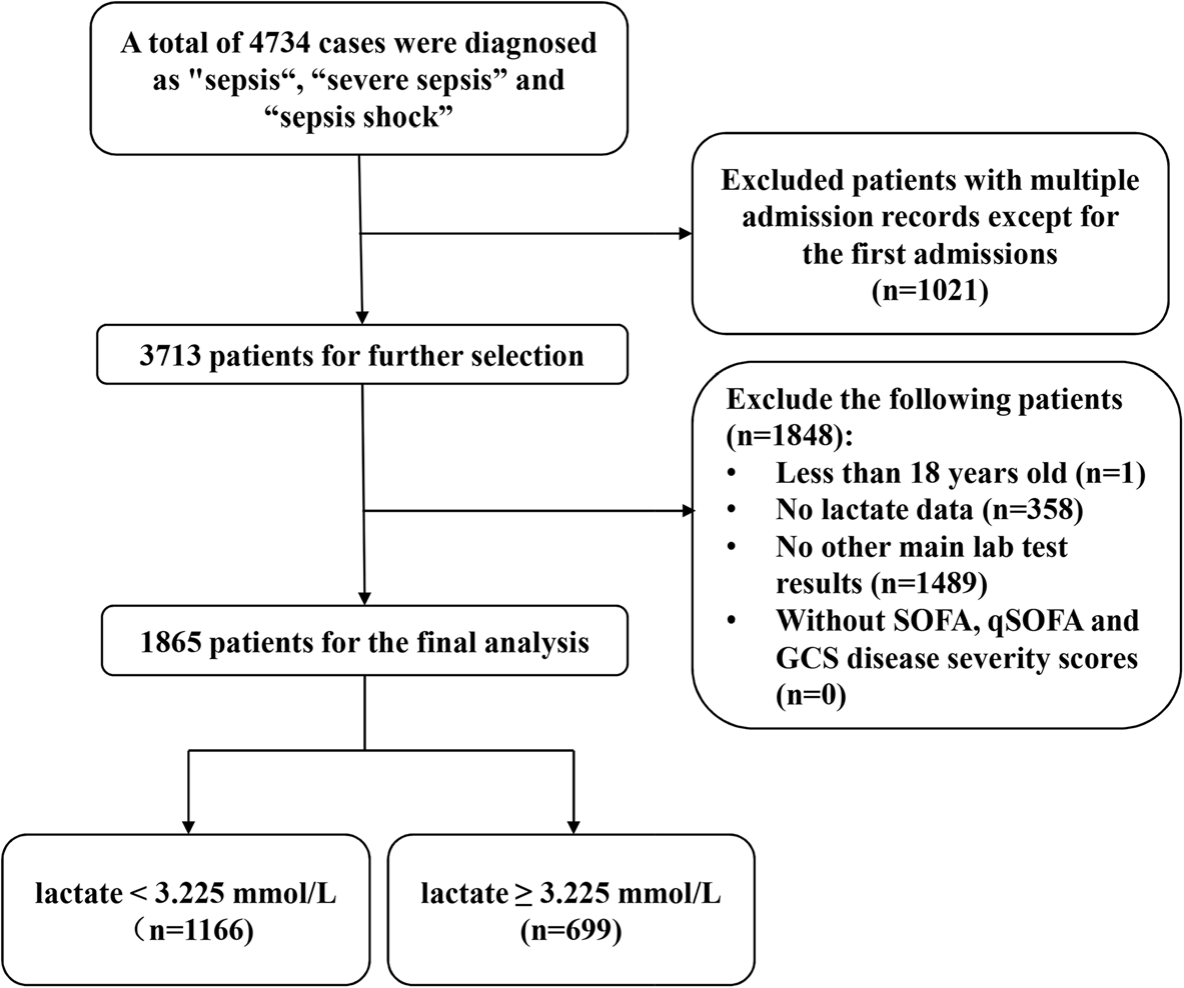
The detailed process of data extraction

We obtained the related information on patients who were diagnosed with “sepsis”, “severe sepsis” and “septic shock” on discharge. A total of 3713 sepsis patients were included. Variables with missing data are common in the MIMIC III database. We excluded patients with missing data (patients without documented lactate or main laboratory tests including hemoglobin, albumin, WBC, bilirubin, BUN, potassium, sodium, bicarbonate, Cr, platelet analyzed in Table 2 in first 24 h from ICU admission were excluded) and patients less than 18 years of age, 1865 patients in our cohort finally met the inclusion criteria in our cohort. The detailed process of data extraction is shown in Fig. 1. We collected the following data: baseline demographic information such as age, sex, weight, and ethnicity; clinical parameters including vital signs, hospital stay, ICU stay, and survival status; laboratory tests and scores on disease scoring systems including Sequential Organ Failure Assessment (SOFA) (Additional file 8: Table S1) [13], quick SOFA (qSOFA) Additional file 9: Table S2) [1, 14], and the Glasgow Coma Scale (GCS) [17]. We retrieved the SQL scripts from the github website (https://github.com/MIT-LCP/mimic-code/tree/master/concepts/severityscores) and used them to calculate the severity scores. The SOFA, qSOFA and GCS scores of the patients were calculated based on the data obtained during the first 24 h of each patient’s ICU’s stay; the scores represent the worst scores during the first 24 h of ICU admission. The lactate level measured during the first 24 h of ICU admission was used in this study. If lactate was measured multiple times in the first 24 h, the average lactate level was used in our study.

### Statistical analysis

Lactate was divided into two groups based on the cut-off value (3.225 mmol/L) which was determined by receiver operating characteristic (ROC) curve analysis [18]. Lactate group 1 included patients with lactate < 3.225 mmol/L, and lactate group 2 included patients with lactate ≥3.225 mmol/L. Other continuous variables were categorized and divided into groups of equal size based on the number of patients. Normally and non-normally distributed continuous variables were summarized as the mean ± SD and as the median with interquartile range (IQR), respectively. The Shapiro-Wilk test and the Kruskal Wallis test were used to assess significant differences. Categorical variables were assessed using chi-square test or Fisher’s exact test. Kaplan-Meier curves were analysed using log-rank tests. The Cox regression model was used to analyse the independent effects of various parameters on 30-day mortality. All the analyses were conducted using R software (version 3.4.3, CRAN), SPSS software (v20.0; IBM, Armonk, NY), MedCalc Statistical Software (v15.2.2; MedCalc Software bvba, Ostend, Belgium) and PASS software (v15; NCSS Statistical Software, Kaysville, Utah, USA); a *P* values< 0.05 represents statistical significance.

## Results

### Baseline characteristics and outcomes

A total of 1865 sepsis patients are included in this study. The baseline characteristics, vital signs, laboratory parameters and outcomes of the patients are summarized in Table 1. The lactate levels of patients in between lactate group 1 and lactate group 2 differ significantly (median 2.05 mmol/L vs. 4.95 mmol/L, *p* < 0.001). Patients with lower lactate have better outcomes (30-day mortality, 33.88% vs. 59.23%; 90-day mortality, 43.57% vs.67.81%; 1-year mortality, 51.46% vs. 71.39%; in-hospital mortality, 34.99% vs. 60.80%; overall mortality, 60.12% vs. 77.97%) and lower SOFA scores (median 7 vs. 11, p < 0.001), and a lower proportion of these patients have qSOFA scores of 2 or more points (84.48% vs. 91.99%). In our cohort, lactate levels show no correlation with severe sepsis rate (96.05% vs. 94.13%, *p* = 0.057). However, a higher proportion of patients with higher lactate levels have septic shock (63.29%vs. 70.24%, *p* = 0.002).

**Table 1:** Baseline characteristics, vital signs, laboratory parameters and outcomes of patients with sepsis

		Lactate group 1	Lactate group 2	Total	P
Number		1166	699	1865	
Baseline variables					
Age		70 (56–80)	66 (55.5–77)	68 (56–78.25)	0.057
Sex (%)	Female	502 (43.05)	300 (42.92)	802 (43.00)	0.955
	Male	664 (56.95)	399 (57.08)	1063 (57.00)	
Ethnicity (%)	White	883 (71.43)	473 (67.67)	1356 (72.71)	< 0.001
	Black	94 (8.06)	76 (10.87)	170 (9.12)	
	Yellow	29 (2.49)	25 (3.58)	54 (2.90)	
	Others	160 (13.72)	125 (17.88)	285 (15.28)	
Admission type (%)	Urgent	22 (1.89)	16 (2.29)	38 (2.04)	0.836
	Emergency	1088 (93.31)	650 (92.99)	1738 (93.19)	
	Elective	56 (4.80)	33 (4.72)	89 (4.77)	
ICU stay (%)	CCU	94 (8.06)	63 (9.01)	157 (84.18)	0.247
	CSRU	65 (5.57)	29 (4.15)	94 (50.40)	
	MICU	705 (60.46)	441 (63.09)	1146 (61.45)	
	SICU	188 (16.12)	114 (16.31)	302 (16.19)	
	TSICU	114 (9.78)	52 (7.44)	166 (8.90)	
Vital signs					
	HR	93 (79–107.5)	101 (82.5–112)	96 (81–109)	< 0.001
	SBP	106 (99–111.5)	104 (95.5–112)	104 (97–112)	< 0.001
	DBP	54 (48–61)	53 (49–61.5)	53.5 (48.75–61)	0.092
	MBP	70 (66–76.5)	68 (64–77)	69.5 (65–77)	0.001
	RR	21 (19–24)	23 (19.5–26)	22 (19–26)	< 0.001
	T	37 (36–37)	37 (36–37)	37 (36–37)	< 0.001
	SpO2	97 (95–99)	97 (94–98)	97 (95–99)	< 0.001
Laboratory parameters (mmol/L)					
	Hemoglobin	10.2 (9.1–12.79)	10.4 (9.2–11.8)	10.2 (9.1–11.6)	0.051
	Albumin	2.7 (2.3–3.1)	2.6 (2.2–3.1)	2.6 (2.2–3.1)	0.220
	WBC	14.2 (9.2–40)	13.45 (7.55–20.43)	14 (8.6–20.2)	0.423
	Bilirubin	0.8 (0.4–1.8)	1.35 (0.6–4.1)	1 (0.5–2.5)	0.001
	BUN	35 (21–57)	36.5 (22.5–78.55)	35.5 (21.5–56)	0.001
	Potassium	4.1 (3.8–4.6)	4.4 (3.9–4.9)	4.2 (3.8–4.8)	< 0.001
	Sodium	138.5 (135–141.38)	138 (134.5–141.5)	138 (135–141.5)	< 0.001
	Bicarbonate	20.5 (18–24)	17.5 (14.5–20.5)	19.5 (16–22.5)	< 0.001
	Cr	1.6 (1–2.8)	1.9 (1.2–3.1)	1.8 (1.08–3)	< 0.001
	Platelet	193.5 (120.25–290.5)	154 (86.5–241.25)	180 (104.5–265.38)	0.300
	Lactate	2.05 (1.53–2.80)	4.95 (3.90–7.35)	3.23 (2.04–4.96)	< 0.001
Score system					
	SAPS II	47 (37–56)	57 (46–69)	50 (40–61)	< 0.001
	SOFA	7 (5–10)	11 (8–13)	8 (6–11)	< 0.001
	GCS	15 (14–15)	15 (13–15)	15 (13–15)	0.685
qSOFA (%)	0	26 (2.23)	10 (0.015)	36 (1.93)	< 0.001
	1	155 (13.29)	46 (6.58)	201 (10.78)	
	2	763 (65.44)	486 (69.53)	1249 (66.97)	
	3	222 (19.04)	157 (22.46)	379 (20.32)	
Outcome (%)					
	30-day mortality	395 (33.88)	414 (59.23)	809 (43.38)	< 0.001
	90-day mortality	508 (43.57)	474 (67.81)	982 (52.65)	< 0.001
	1-year mortality	600 (51.46)	499 (71.39)	1099 (58.93)	< 0.001
	Hospital mortality	408 (34.99)	425 (60.80)	833 (44.66)	< 0.001
	Severe sepsis (%)	1120 (96.05)	658 (94.13)	1778 (95.34)	0.057
	Septic shock (%)	738 (63.29)	491 (70.24)	1229 (65.90)	0.002

ICU, intensive care unit; SICU, surgical intensive care unit; CCU, cardiac care unit; CSRU, cardiac surgery recovery unit; MICU, medical intensive care unit; TSICU, Trauma surgical intensive care unit; GCS, Glasgow Coma Scale; SBP, systolic blood pressure; DBP, diastolic blood pressure; MBP, mean blood pressure; HR, heart rate; RR, respiratory rate; T, temperature; WBC, white blood cell; BUN, blood urea nitrogen; Cr, creatinine. *P* < 0.05 means significant different

### Lactate is an independent prognostic predictor in sepsis patients

**Fig. 2:**
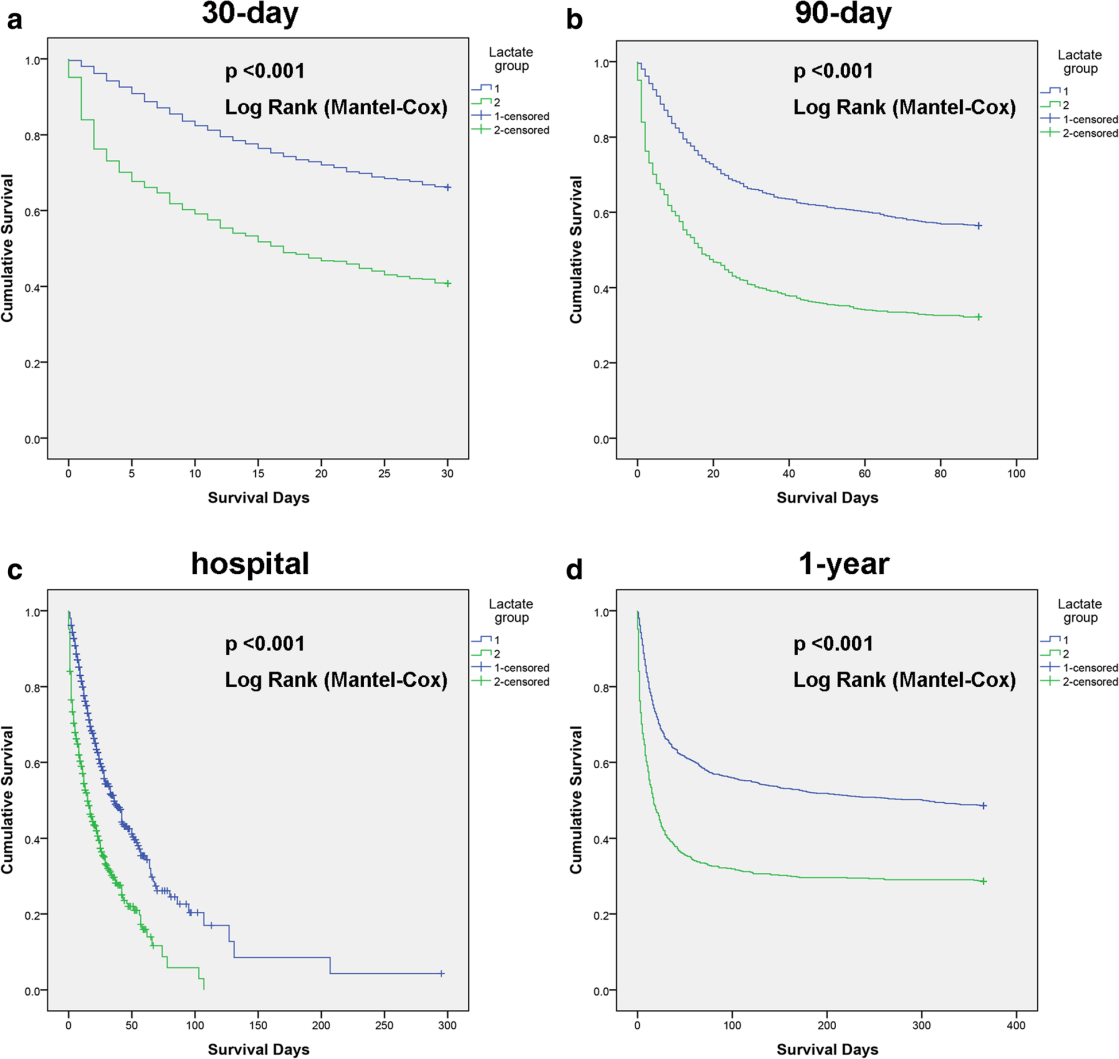
Probability of mortality curve for the patient with sepsis by lactate levels. **a**. 30-day mortality; **b**. 90-day mortality; **c**. hospital mortality; **d**. 1-year mortality. *P* values were calculated using log Rank-Mantel. P< 0.05 means statistically significant

Survival analysis was conducted to explore the impact of lactate on prognosis. Notably, patients in the lower lactate group had better short-term and long-term survival rates. Patients in the higher lactate group had increased 30-day, 90-day, 1-year and in-hospital mortality (Fig. 2). Furthermore, we performed univariate analysis of baseline variables (age, sex, ethnicity, admission type, and ICU type) and laboratory tests (haemoglobin, albumin, WBC, bilirubin, BUN, potassium, sodium, bicarbonate, Cr, platelets, and lactate). Age, sex, admission type, ICU type, albumin, bilirubin, BUN, potassium, bicarbonate, Cr, platelets and lactate were analysed in the univariate analysis, and the factors significantly correlated with OS were adjusted for multivariate analysis. According to the results, lactate remained an independent prognostic factor for sepsis (Table 2). The Kaplan-Meier survival curves of patients with different lactate levels in different ICU types are shown in Additional file 1: Figure S1, Additional file 2: Figure S2, Additional file 3: Figure S3, Additional file 4: Figure S4. Additional file 5: Figure S5. The AUROC for qSOFA, SOFA, lactate and the AUROC for qSOFA, SOFA and lactate in different ICU types are shown in Additional file 6: Table S3 and Additional file 7: Table S4.

**Table 2:** Univariate and multivariate analysis of risk factors to 30-day mortality

	Univariate analysis				Multivariate analysis			
	P	Hazard Ratio	95.0% CI		P	Hazard Ratio	95.0% CI	
			Lower	Upper			Lower	Upper
Age	< 0.001	1.564	1.360	1.799	< 0.001	1.637	1.397	1.917
Sex	0.170	1.103	0.959	1.269	0.027	1.076	1.008	1.148
Ethnicity	0.003	1.094	1.031	1.161				
Admission type	0.001	0.624	0.474	0.820	0.015	0.698	0.522	0.934
ICU type	0.007	0.906	0.843	0.973				
Hemoglobin	0.030	0.858	0.748	0.985				
Albumin	0.045	0.862	0.746	0.997	0.009	0.813	0.697	0.949
WBC	0.896	1.009	0.879	1.158				
Bilirubin	< 0.001	1.576	1.354	1.836	< 0.001	1.366	1.160	1.607
BUN	< 0.001	1.816	1.576	2.093	0.003	1.285	1.086	1.520
Potassium	< 0.001	1.672	1.453	1.924	< 0.001	1.578	1.342	1.855
Sodium	0.619	0.966	0.841	1.108				
Bicarbonate	< 0.001	0.635	0.553	0.730	0.005	0.793	0.674	0.932
Cr	< 0.001	1.649	1.434	1.897				
Platelet	< 0.001	0.703	.612	0.808	0.004	0.789	0.671	0.928
Lactate	< 0.001	2.304	2.007	2.646	< 0.001	1.936	1.649	2.271

**Fig. 3:**
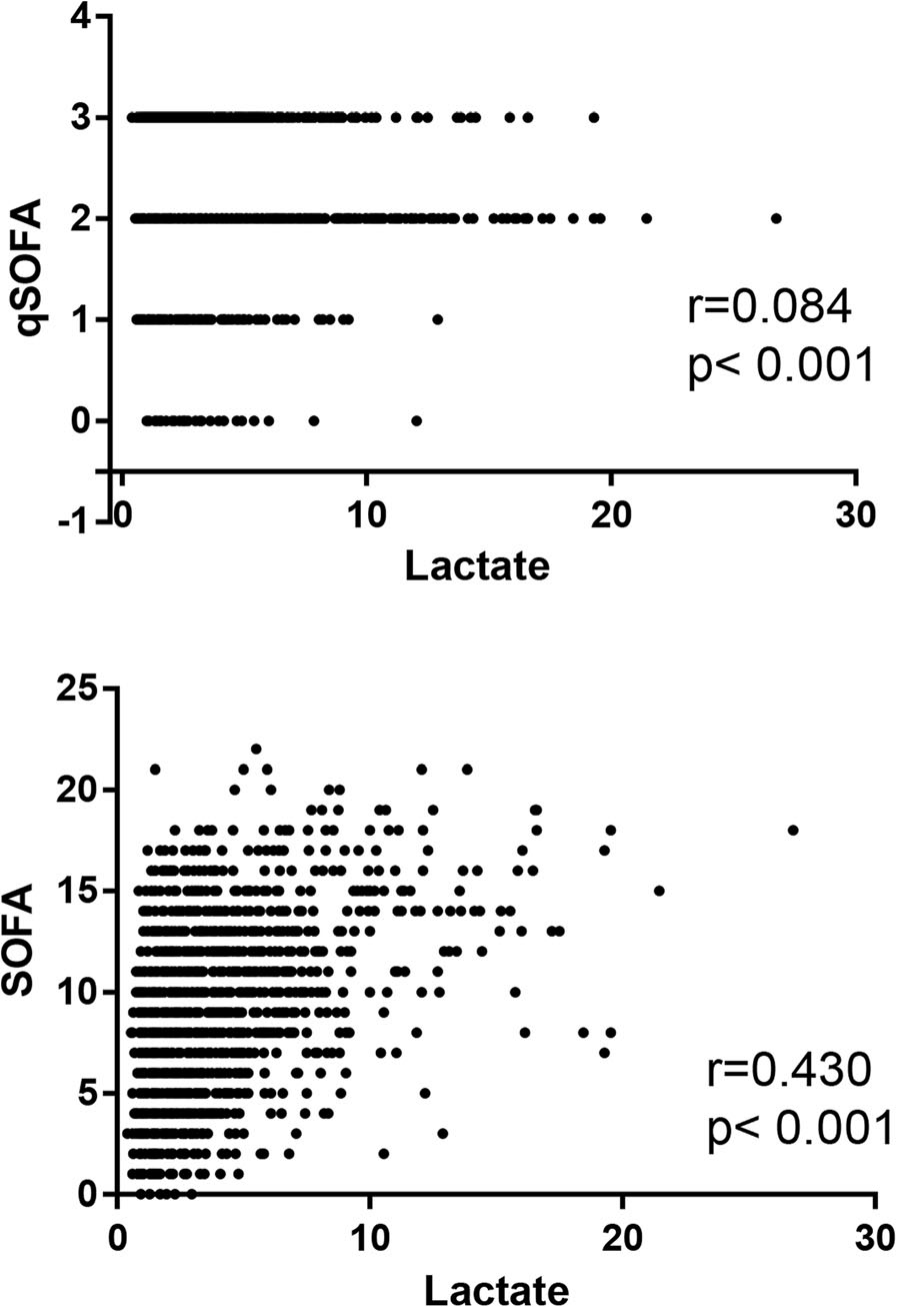
The association between lactate and SOFA score (*r* = 0.43, *P* = 0). Pearson analysis was performed

SOFA is often used to evaluate organ dysfunction and is correlated with mortality [1]. Figure 3 illustrates the relationship between lactate and qSOFA/SOFA score (*r* = 0.084, *p* < 0.001; *r* = 0.430, p < 0.001, respectively) in patients with sepsis. Higher lactate levels correlate with higher qSOFA/SOFA scores.

### Comparison of lactate to SOFA and qSOFA scores

**Fig. 4:**
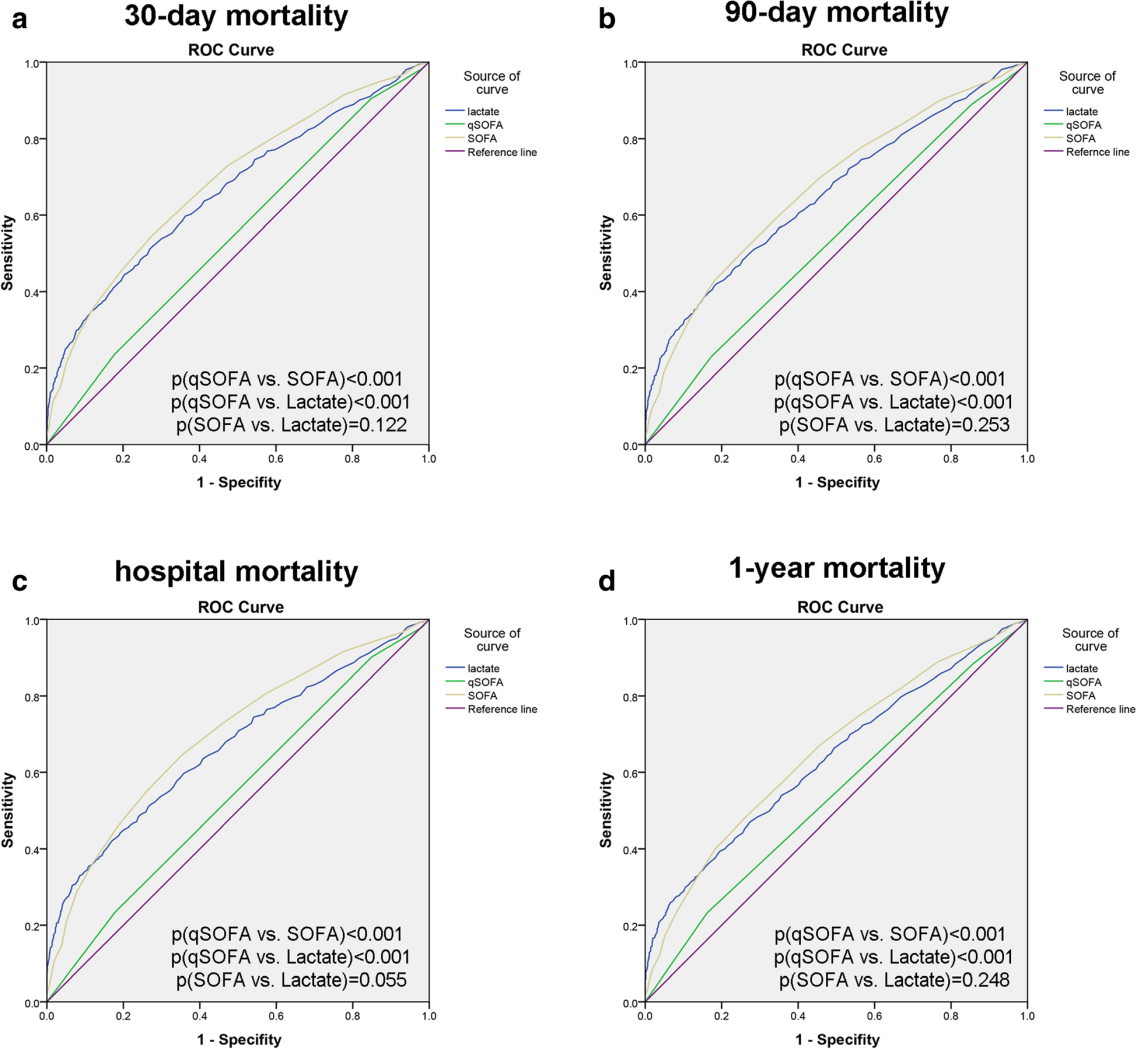
Receiver operating characteristic curves of lactate for predicting mortality. **a**. 30-day mortality; **b**. 90-day mortality; **c**. hospital mortality; **d**. 1-year mortality

The Sepsis-3 criteria recommended qSOFA based on its simple bedside criteria and its ability to facilitate prompt identification of suspected infectious adult patients who are likely to have poor outcomes [1]. Discrimination of 30-day mortality (Fig. 4; Table 3), which was assessed by the AUROC, was significantly higher for lactate (AUROC, 0.664) than for qSOFA (AUROC, 0.547). The discriminative power of lactate was similar to that of SOFA (AUROC, 0.686). Moreover, the same trends were observed for 90-day mortality, hospital mortality and 1-year mortality (Fig. 4; Table 3).

**Table 3:** AUROC of lactate, qSOFA, SOFA for mortality

	Area Under ROC curve			
	30-day mortality (95%CI)	90-day mortality (95%CI)	hospital mortality (95%CI)	1-year mortality (95%CI)
Lactate	0.664 (0.639,0.689)	0.656 (0.632,0.681)	0.668 (0.643,0.693)	0.636 (0.611,0.661)
qSOFA	0.547 (0.521,0.574)	0.539 (0.513,0.565)	0.544 (0.518,0.571)	0.541 (0.514,0.567)
SOFA	0.686 (0.661,0.710)	0.673 (0.648,0.697)	0.695 (0.671,0.719)	0.653 (0.628,0.677)
Lactate+qSOFA	0.672 (0.647–0.697)	0.661 (0.636–0.685)	0.674 (0.650–0.699)	0.640 (0.616–0.665)

Next, we selected the cut-off values of lactate, qSOFA, SOFA that were highly sensitive and specific in predicting short-term and long-term mortality, through ROC curve analysis. The sensitivities, specificities, positive/negative predictive values and positive/negative likelihood ratios (LR+/−) of lactate for mortality are shown in Table 4. The values of LR+/− were partiallly consistent with the results reported in a previous study [19].

**Table 4:** Diagnostic sensitivity, specificity, predictive values and positive/negative likelihood ratios of lactate, qSOFA, SOFA for mortality

		Sensitivity	Specifity	PPV	NPV	LR+	LR-
	Lactate	0.512	0.730	0.592	0.339	1.453	0.512
30-day mortatily	qSOFA	0.236	0.822	0.496	0.416	0.817	0.481
	SOFA	0.545	0.725	0.603	0.325	1.521	0.480
	Lactate	0.414	0.820	0.669	0.361	2.023	0.566
90-day mortatily	qSOFA	0.230	0.827	0.594	0.509	1.159	0.837
	SOFA	0.510	0.740	0.685	0.424	2.178	0.737
	Lactate	0.421	0.829	0.666	0.360	1.994	0.563
hospital mortatily	qSOFA	0.234	0.822	0.515	0.429	0.856	0.529
	SOFA	0.645	0.647	0.595	0.307	1.475	0.443
	Lactate	0.394	0.804	0.757	0.523	3.117	1.097
1-year mortatily	qSOFA	0.232	0.838	0.673	0.568	1.478	1.174
	SOFA	0.482	0.738	0.725	0.498	2.637	0.993

PPV, Positive predictive value; NPV, Negative predictive value; LR+, Positive likelihood ratio; LR-, Negative likelihood ratio

## Discussion

Multiple studies have explored the association between lactate levels and prognosis in critically ill patients. Lower lactate levels, and even levels in the normal range, were reported to be related to lower severity of sepsis [12]. *Wacharasint* et al demonstrated that patients with lactate levels in the normal-range (between 1.4 and 2.3 mmol/L) had markedly increasing risk of organ failure and higher mortality compared with patients who had lactate levels less than 1.4 mmol/L in two cohorts [12]. Sepsis-3 recommended serum lactate level > 2 mmol/L as a major criterion for the clinically identification of septic shock [1]. A lactate concentration greater than 4 mmol/L was described as having a specificity of 96% in predicting hospital mortality in non-hypotensive patients [20]. A retrospective study that included 7155 ICU patients showed that a significant association between lactate concentration and increased hospital mortality was first detectable at the time weighted lactate concentration was greater than 0.75 mmol/L [7]. Lactate has also used to guide resuscitation efficacy [21]. At the same time, the use of qSOFA and SOFA has been endorsed by professional societies worldwide.

Recently, *Simpson* raised concerns that reliance on qSOFA or SOFA criteria may lead to delayed diagnosis and intervention in cases of serious infection [22]. Furthermore, a systematic review and meta-analysis demonstrated that qSOFA was poorly sensitive and moderately specific for the risk of death [23]. *Ho* [24] found that qSOFA had modest mortality predictive ability in both septic and non-septic patients in a prospective study. When combined with lactate, qSOFA showed predictive ability comparable to that of SOFA. An observational cohort study that included patients with infection who were admitted in the emergency department demonstrated poor performance of qSOFA in predicting mortality [25]. Therefore, we compared mortality prediction by serum lactate to mortality prediction by SOFA and qSOFA. First, we found that lactate was positively associated with qSOFA/SOFA scores (Fig. 3) and prognosis (Fig. 2). Next, lactate was shown to be an independent prognostic predictor by Cox regression model analysis (Table 2). Lactate showed superior prognostic accuracy for short-term and long-term mortality compared to qSOFA, and the predictive validity of lactate was similar to that of SOFA (Table 3). The AUROC of SOFA and of qSOFA combined with lactate was similar to the AUROC reported in a previous study in septic patients [24]. However, in our cohort, qSOFA showed a capacity to predict mortality in patients with an AUROC lower than that found in Ho’s study [24]. qSOFA had far less sensitivity, but similar specificity to lactate and SOFA (Table 4). Combining lactate with qSOFA yielded a predictive ability close to that of the SOFA score alone (Table 3).

Some studies revealed that the qSOFA score is associated with hospital mortality and that it present higher prognostic accuracy than the SIRS score in adults with suspected infection [14, 26]. The task force of Sepsis-3 suggested using qSOFA to assess organ dysfunction due to its convenience, rapid performance and repeatability. Lactate was not included as an indicator of illness severity. However, a recent study suggested that lactate reduction in the first day of ICU admission is correlated with improved outcome of septic patients regardless of the haemodynamic status [27]. Our study revealed that the sensitivity of qSOFA was low, although it showed relatively high specificity. Lactate testing is a simple, inexpensive, and reasonably sensitive tool for predicting mortality and is more stable than qSOFA when sedation is used. Lactate levels correlated significantly with the SOFA scores. This is consistent with existing studies [28–30]. High lactate can be considered a warning signal for organ dysfunction and is a signal for urgent medical intervention. In fact, high sensitivity may be superior to specificity in the context of fatal sepsis.

Although lactate was reported to guide resuscitation efficacy [21], multicentre, randomized trials indicated that fluid resuscitation was not beneficial to septic shock patients with or without hyperlactataemia [31, 32]. Moreover, fluid resuscitation and lactate clearance might be harmful when hyperlactataemia is not caused by hypoperfusion [5]. However, high lactate levels should be interpreted in accordance with the patient’s specific circumstances.

Our study has several limitations. This study is not prospective but retrospective. The inherent bias of retrospective studies could not be avoided. Lactate demonstrated low sensitivity and moderate specificity for short- and long-term mortality in septic patients. Lactate alone would not be a good predictive test of mortality. Approximately half of the adult sepsis patients in the database were excluded due to lack of laboratory testing; the significant number of excluded patients may affect our conclusion. In addition, because the data were collected from different types of ICUs, the heterogeneity of treatment further complicates the interpretation of the results. Previous studies have established that early lactate levels are associated with organ dysfunctions and mortality in the ICU and emergency departments [10, 33–35]. Delays in lactate measurement are correlated with increased mortality of septic patients [36]. However, in our cohort, the time that elapsed between the first lactate measurement and the onset of sepsis was inconsistent, and the time at which the patient’s lactate was measured in the first 24 h was also inconsistent. Although we used the 24-h average lactate levels to eliminate the bias, the levels might be influenced by resuscitation of the patients, and the average lactate levels may not reflect the true resuscitation state of the patients. Additionally, interventions that might affect SOFA scores, qSOFA scores and prognosis were not included in the study, and lack of these data may affect the analysis. It is difficult to assess the impact of the change in the definition of sepsis on our results due to the de-identification process of the data in the database. Prospective studies need to be conducted, and better outcome predictors need to be further explored.

## Conclusions

In addition to its simplicity and accuracy, lactate is a better prognostic factor than qSOFA and SOFA in adult patients with sepsis. Further study is needed given that this work is based on retrospective data and that the timing of lactate determination in this study may affect results obtained.

## Additional files

Additional file 1: **Figure S1.** Receiver operating characteristic curves of lactate for predicting mortality in CCU patients. (TIF 280 kb)

Additional file 2: **Figure S2.** Receiver operating characteristic curves of lactate for predicting mortality in CSRU patients. (TIF 277 kb)

Additional file 3: **Figure S3.** Receiver operating characteristic curves of lactate for predicting mortality in MICU patients. (TIF 317 kb)

Additional file 4: **Figure S4.** Receiver operating characteristic curves of lactate for predicting mortality in SICU patients. (TIF 312 kb)

Additional file 5: **Figure S5.** Receiver operating characteristic curves of lactate for predicting mortality in TSICU patients. (TIF 276 kb)

Additional file 6: **Table S3.** Sequential [Sepsis-Related] Organ Failure Assessment (SOFA) Score (DOCX 14 kb)

Additional file 7: **Table S4.** qSOFA (Quick SOFA) Criteria (DOCX 12 kb)

Additional file 8: **Table S1**. Areas under the ROC curves for qSOFA, SOFA, lactate (DOCX 13 kb)

Additional file 9: **Table S2.** Comparison of AUROC for qSOFA, SOFA and lactate in different ICU types (DOCX 14 kb)

## Abbreviations

AUCROC: Area under the receiver operating characteristic curve
BIDMC: Beth Israel Deaconess Medical Center
BUN: Blood urea nitrogen
CCU: Cardiac care unit
CI: Confidence interval
Cr: Creatinine
CSRU: Cardiac surgery recovery unit
DBP: Diastolic blood pressure
GCS: Glasgow Coma Scale
HR: Hazard ratio
ICU: Intensive care unit
IQR: Interquartile range
MBP: Mean blood pressure
MICU: Medical intensive care unit
MIMIC: Medical Information Mart for Intensive Care
MIT: Massachusetts Institute of Technology
N: Number
qSOFA: quick SOFA
ROC: Receiver operating curve
RR: Respiratory rate
SBP: Systolic blood pressure
Sepsis-3: The Third International Consensus Definitions for Sepsis and Septic Shock
SICU: Surgical intensive care unit
SOFA: Sequential Organ Failure Assessment
SPO_2_: Percutaneous oxygen saturation
SQL: Structure query language
TSICU: Trauma surgical intensive care unit
WBC: White blood cell
