# Supplementary figures and images for: Prognostic accuracy of the serum lactate level, the SOFA score and the qSOFA score for mortality among adults with Sepsis
Source: Scand J Trauma Resusc Emerg Med. 2019 Apr 30;27:51. doi: 10.1186/s13049-019-0609-3 (PMC6492372; doi:10.1186/s13049-019-0609-3)

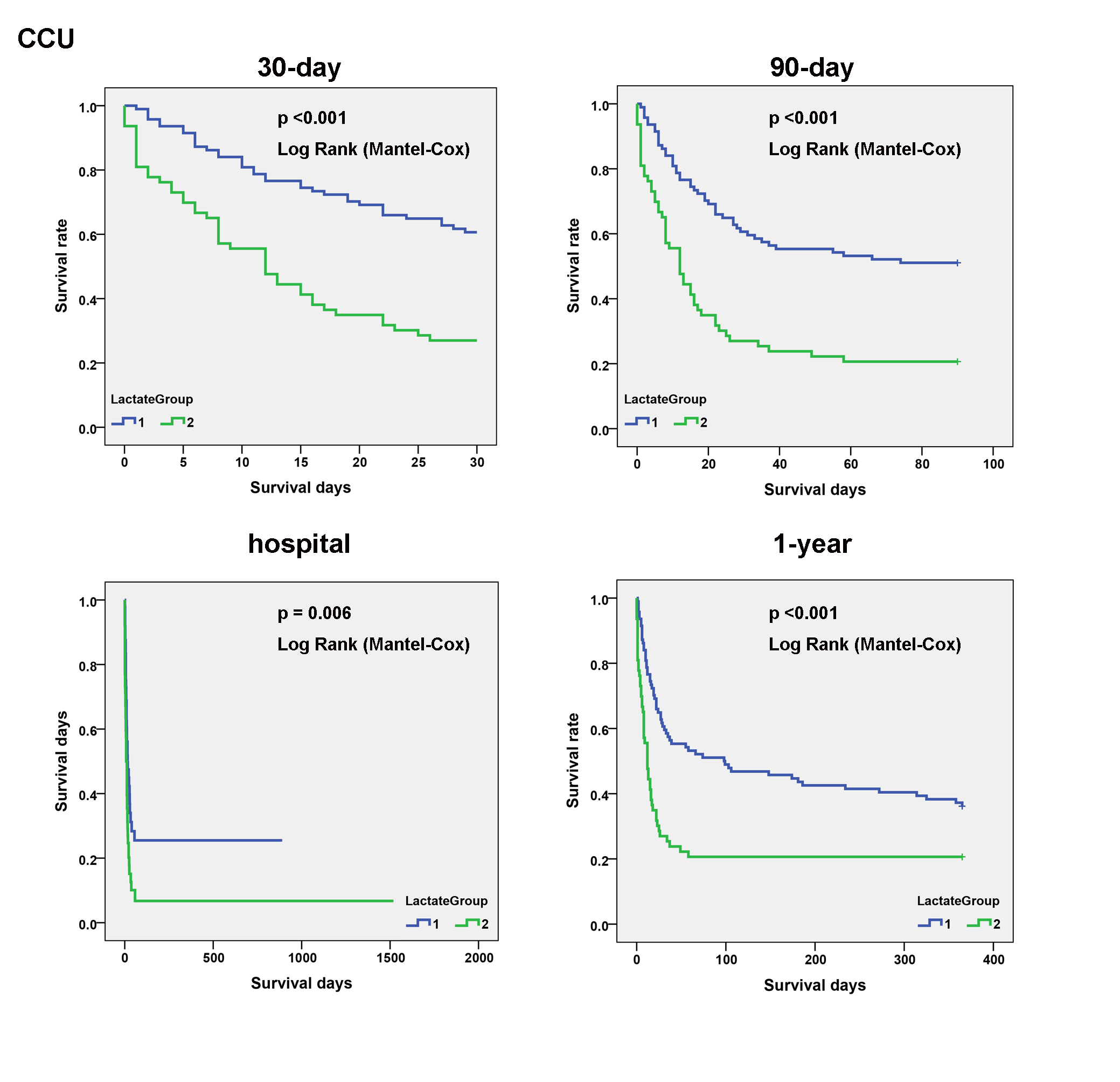

Supplement: Supplementary file 1 — Figure S1. Receiver operating characteristic curves of lactate for predicting mortality in CCU patients. (TIF 280 kb) [file 13049_2019_609_MOESM1_ESM.tif]

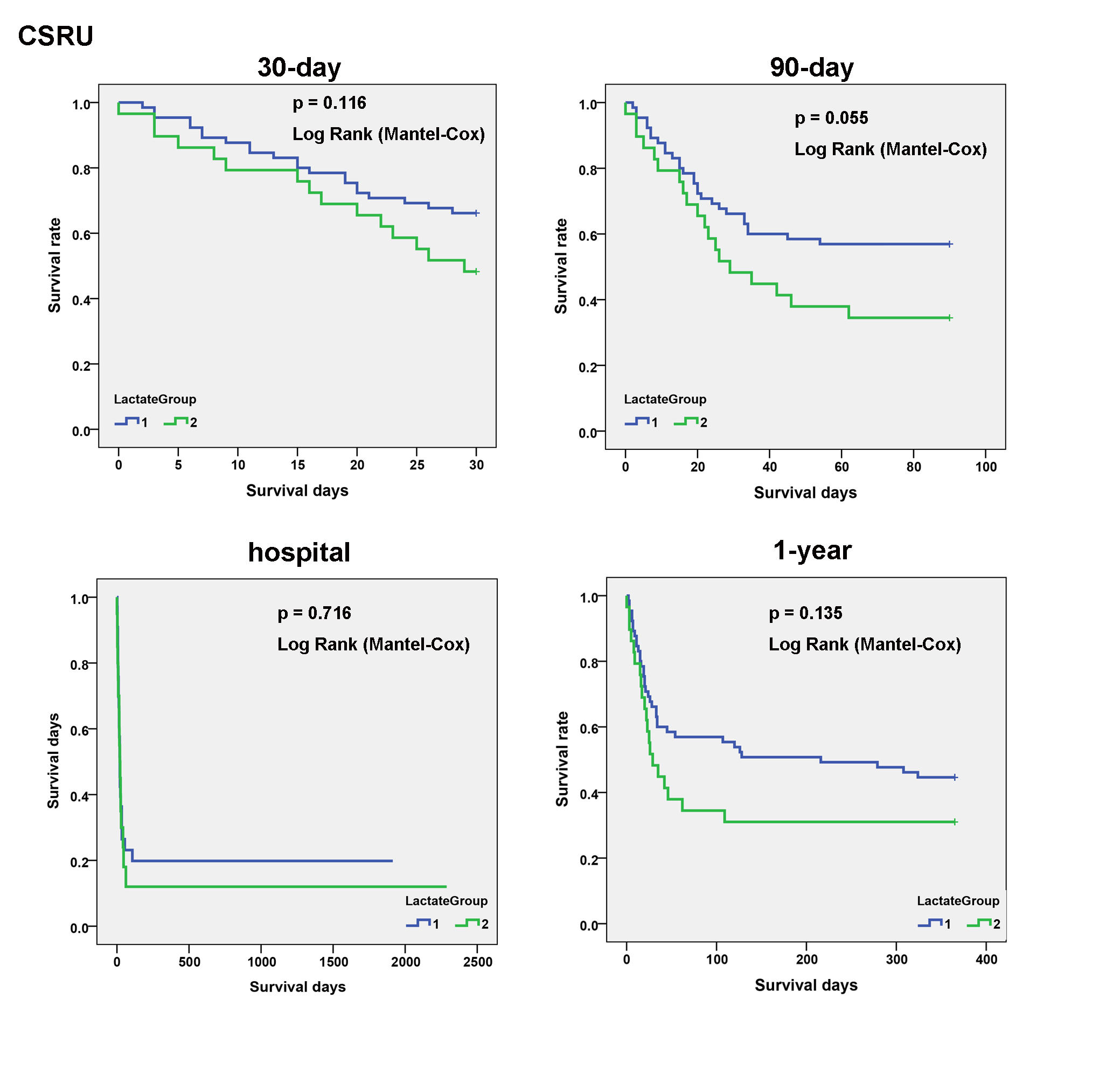

Supplement: Supplementary file 2 — Figure S2. Receiver operating characteristic curves of lactate for predicting mortality in CSRU patients. (TIF 277 kb) [file 13049_2019_609_MOESM2_ESM.tif]

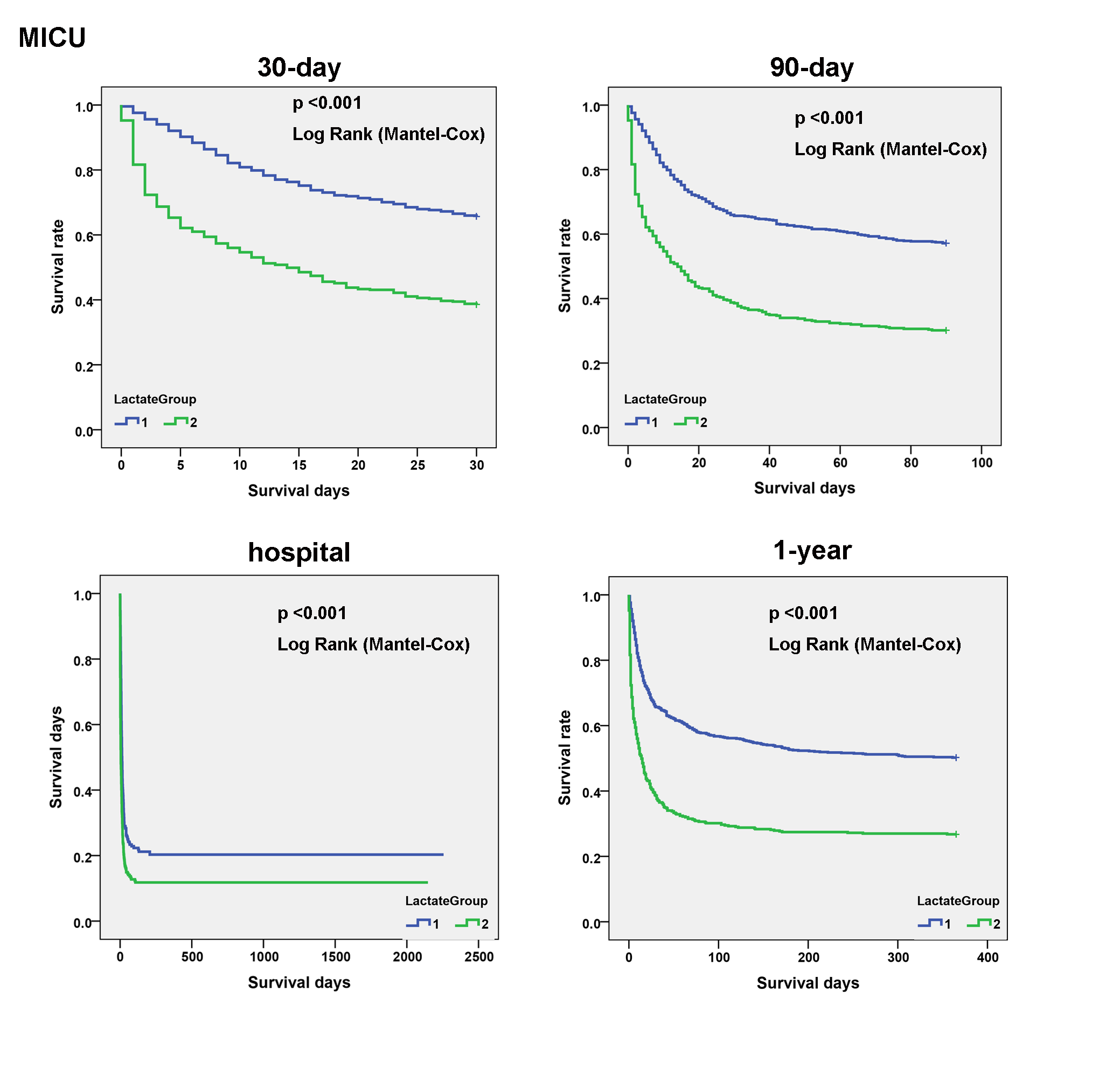

Supplement: Supplementary file 3 — Figure S3. Receiver operating characteristic curves of lactate for predicting mortality in MICU patients. (TIF 317 kb) [file 13049_2019_609_MOESM3_ESM.tif]

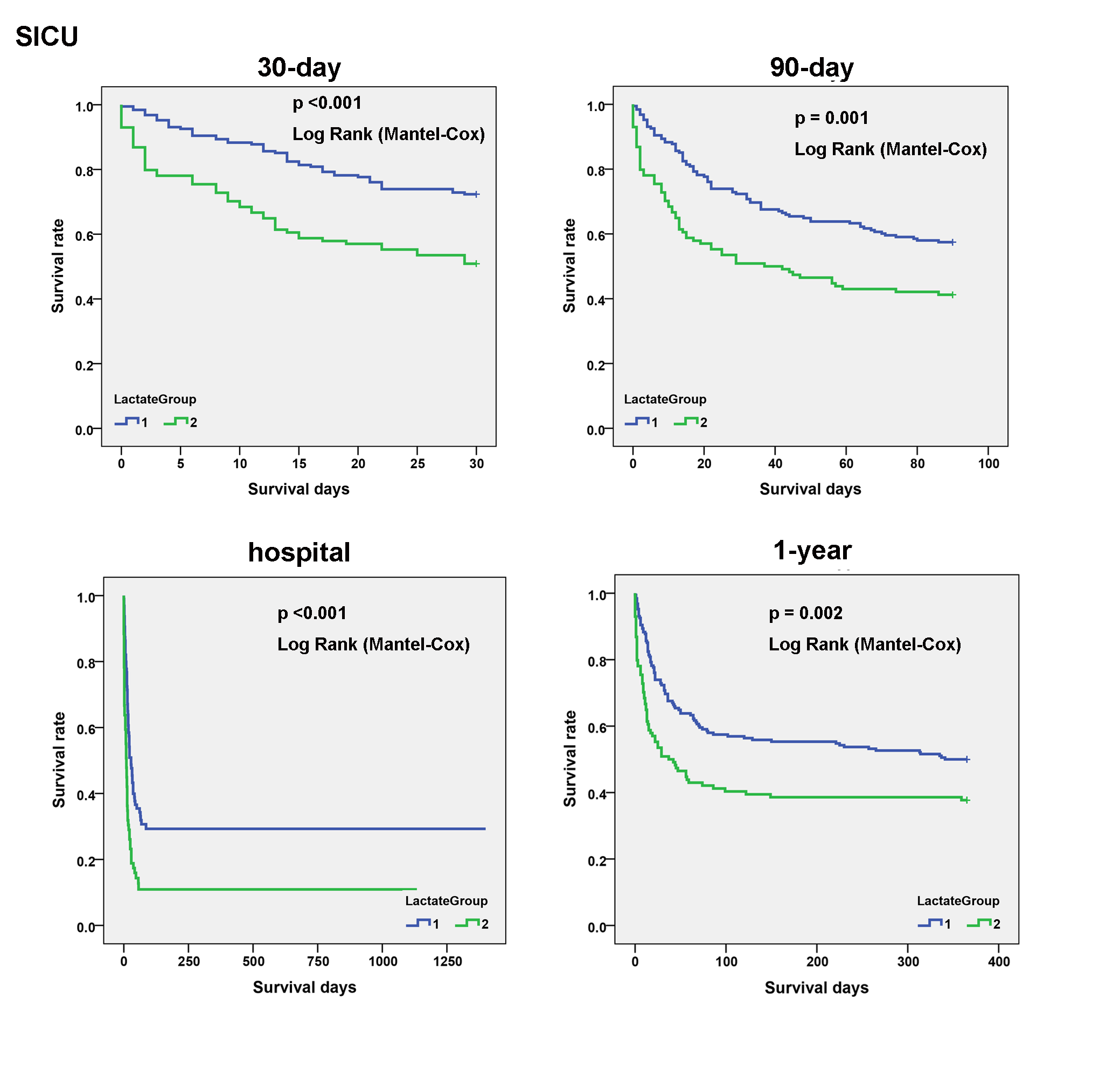

Supplement: Supplementary file 4 — Figure S4. Receiver operating characteristic curves of lactate for predicting mortality in SICU patients. (TIF 312 kb) [file 13049_2019_609_MOESM4_ESM.tif]

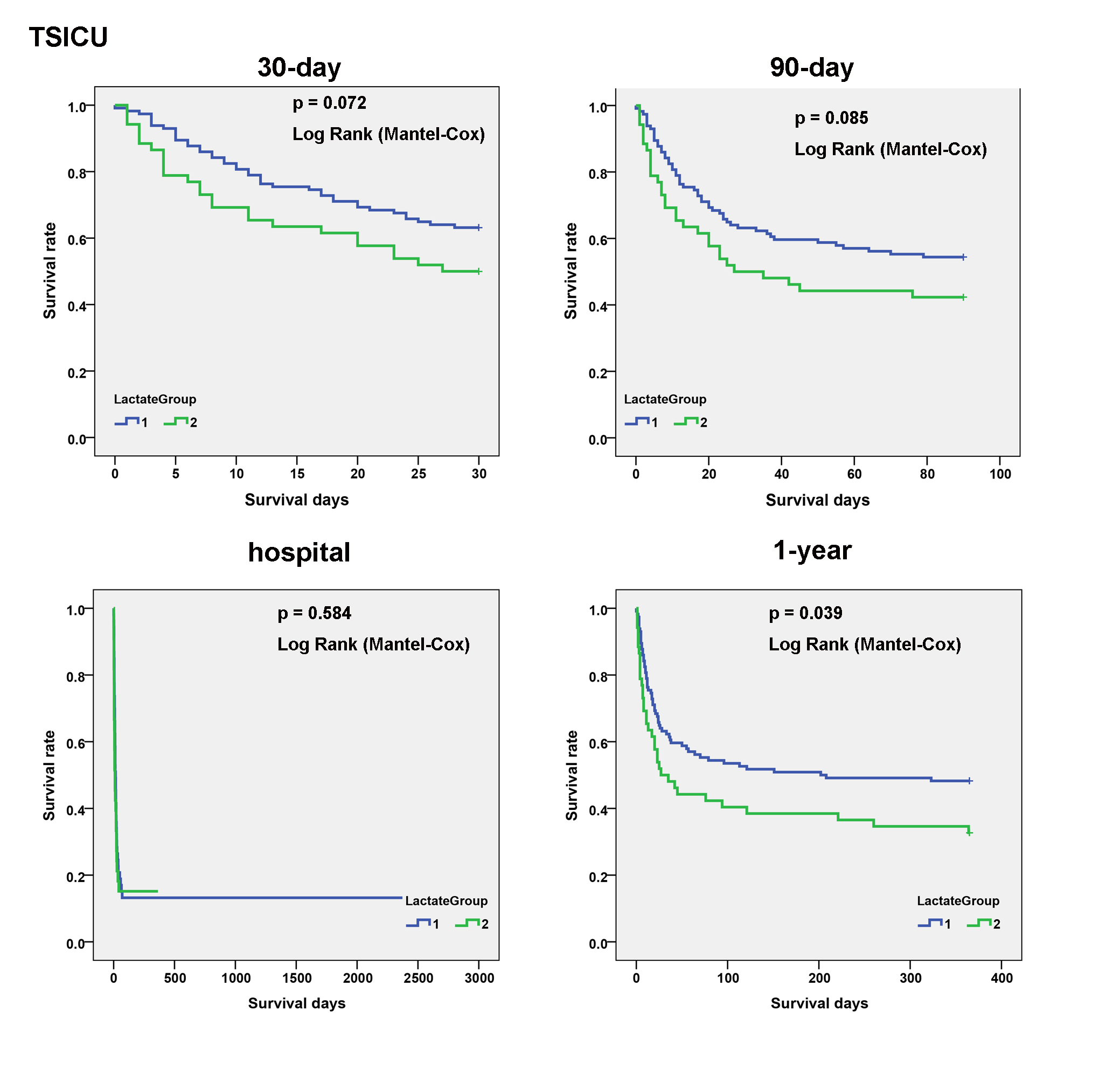

Supplement: Supplementary file 5 — Figure S5. Receiver operating characteristic curves of lactate for predicting mortality in TSICU patients. (TIF 276 kb) [file 13049_2019_609_MOESM5_ESM.tif]
