# Supplementary material for: Prognostic accuracy of the serum lactate level, the SOFA score and the qSOFA score for mortality among adults with Sepsis
Source: Scand J Trauma Resusc Emerg Med. 2019 Apr 30;27:51. doi: 10.1186/s13049-019-0609-3 (PMC6492372; doi:10.1186/s13049-019-0609-3)
Supplement: Supplementary file 6 — Table S3. Sequential [Sepsis-Related] Organ Failure Assessment (SOFA) Score (DOCX 14 kb) [file 13049_2019_609_MOESM6_ESM.docx]

Supplementary Table 3 Sequential [Sepsis-Related] Organ Failure Assessment (SOFA) Score

|  | Score |  |  |  |  |
| --- | --- | --- | --- | --- | --- |
| **System** | 0 | 1 | 2 | 3 | 4 |
| **Respiration** |  |  |  |  |  |
| PaO2/FIO2, mm Hg | ≥400 (53.3) | <400 (53.3) | <300 (40) | <200 (26.7) with | <100 (13.3) with |
| (kPa) |  |  |  | respiratory support | respiratory support |
| **Coagulation** |  |  |  |  |  |
| Platelets, ×103/µL | ≥150 | <150 | <100 | <50 | <20 |
| **Liver** |  |  |  |  |  |
| Bilirubin, mg/dL (µmol/L) | <1.2 (20) | 1.2–1.9 (20–32) | 2.0–5.9 (33–101) | 6.0–11.9 (102–204) | >12.0 (204) |
| **Cardiovascular** | MAP ≥70 mm Hg | MAP <70 mm Hg | Dopamine <5 or | Dopamine 5.1–15 | Dopamine >15 or |
|  |  |  | dobutamine (any dose) ^a^ | or epinephrine ≤0.1 | epinephrine >0.1 |
|  |  |  |  | or norepinephrine ≤0.1^a^ | or norepinephrine >0.1^a^ |
| **Central nervous system** |  |  |  |  |  |
| Glasgow Coma Scale | 15 | 13–14 | 10–12 | 6–9 | <6 |
| score |  |  |  |  |  |
| **Renal** |  |  |  |  |  |
| Creatinine, mg/dL | <1.2 (110) | 1.2–1.9 (110–170) | 2.0–3.4 (171–299) | 3.5–4.9 (300–440) | >5.0 (440) |
| (µmol/L) |  |  |  |  |  |
| Urine output, mL/d |  |  |  | <500 | <200 |

^a^ Catecholamine doses are given as µg/kg/min for at least 1 hour
