# Supplementary material for: Prognostic accuracy of the serum lactate level, the SOFA score and the qSOFA score for mortality among adults with Sepsis
Source: Scand J Trauma Resusc Emerg Med. 2019 Apr 30;27:51. doi: 10.1186/s13049-019-0609-3 (PMC6492372; doi:10.1186/s13049-019-0609-3)
Supplement: Supplementary file 7 — Table S4. qSOFA (Quick SOFA) Criteria (DOCX 12 kb) [file 13049_2019_609_MOESM7_ESM.docx]

Supplementary Table 4 qSOFA (Quick SOFA) Criteria

| Respiratory rate ≥22/min |
| --- |
| Altered mentation |
| Systolic blood pressure ≥100 mm Hg |
