# Supplementary material for: Prognostic accuracy of the serum lactate level, the SOFA score and the qSOFA score for mortality among adults with Sepsis
Source: Scand J Trauma Resusc Emerg Med. 2019 Apr 30;27:51. doi: 10.1186/s13049-019-0609-3 (PMC6492372; doi:10.1186/s13049-019-0609-3)
Supplement: Supplementary file 8 — Table S1. Areas under the ROC curves for qSOFA, SOFA, lactate (DOCX 13 kb) [file 13049_2019_609_MOESM8_ESM.docx]

Supplementary Table 1 Areas under the ROC curves for qSOFA, SOFA, lactate

| **Area Under ROC curve** | | | | | |
| --- | --- | --- | --- | --- | --- |
|  | **CCU** | **CSRU** | **MICU** | **SICU** | **TSICU** |
| qSOFA  95% CI | 0.483  (0.393,0.574) | 0.553  (0.525,0.580) | 0.553  (0.525,0.581) | 0.556  (0.489,0.623) | 0.541  (0.487,0.594) |
|  |  |  |  |  |  |
| SOFA  95% CI | 0.660  (0.575,0.745) | 0.689  (0.664,0.715) | 0.694  (0.668,0.720) | 0.667  (0.599,0.736) | 0.637  (0.584,0.691) |
|  |  |  |  |  |  |
| Lactate  95% CI | 0.697  (0.615,0.778) | 0.661  (0.635,0.668) | 0.663  (0.636,0.690) | 0.686  (0.622,0.750) | 0.661  (0.610,0.712) |

CCU: cardiac care unit; CSRU: cardiac surgery recovery unit; MICU: medical intensive care unit; SICU: surgical intensive care unit; TSICU, Trauma surgical intensive care unit.
