# Supplementary material for: Prognostic accuracy of the serum lactate level, the SOFA score and the qSOFA score for mortality among adults with Sepsis
Source: Scand J Trauma Resusc Emerg Med. 2019 Apr 30;27:51. doi: 10.1186/s13049-019-0609-3 (PMC6492372; doi:10.1186/s13049-019-0609-3)
Supplement: Supplementary file 9 — Table S2. Comparison of AUROC for qSOFA, SOFA and lactate in different ICU types (DOCX 14 kb) [file 13049_2019_609_MOESM9_ESM.docx]

Supplementary Table 2 Comparison of AUROC for qSOFA, SOFA and lactate in different ICU types

|  | CCU |  |  | CSRU |  |  | MICU |  |  | SICU |  |  | TSICU |  |  |
| --- | --- | --- | --- | --- | --- | --- | --- | --- | --- | --- | --- | --- | --- | --- | --- |
|  | AUROC | SE | P | AUROC | SE | P | AUROC | SE | P | AUROC | SE | P | AUROC | SE | P |
| qSOFA | 0.517 | 0.038 | 0.954 | 0.543 | 0.048 | 0.629 | 0.553 | 0.014 | 0.302 | 0.556 | 0.027 | 0.350 | 0.514 | 0.036 | ref. |
| SOFA | 0.660 | 0.043 | 0.239 | 0.611 | 0.059 | 0.744 | 0.712 | 0.015 | 0.008 | 0.667 | 0.035 | 0.157 | 0.587 | 0.044 | ref. |
| Lactate | 0.660 | 0.043 | 0.628 | 0.631 | 0.062 | 0.990 | 0.664 | 0.016 | 0.470 | 0.686 | 0.033 | 0.309 | 0.630 | 0.044 | ref. |

CCU: cardiac care unit; CSRU: cardiac surgery recovery unit; MICU: medical intensive care unit; SICU: surgical intensive care unit; TSICU, Trauma surgical intensive care unit. ref., reference, the lowest AUROC of qSOFA/SOFA/lactate in different ICU types was considered as reference.
